# 3-pyridyl inhibitors with novel activity against *Trypanosoma cruzi* reveal *in vitro* profiles can aid prediction of putative cytochrome P450 inhibition

**DOI:** 10.1038/s41598-018-22043-z

**Published:** 2018-03-20

**Authors:** Melissa L. Sykes, Vicky M. Avery

**Affiliations:** Discovery Biology, Griffith Institute for Drug Discovery, Griffith University, Nathan, Queensland Australia

## Abstract

Using high throughput, high-content imaging, a proprietary library was screened against intracellular *Trypanosoma cruzi* amastigotes to identify compounds with novel activity against the parasite. Five inhibitors were discovered, which did not clear all of the parasites from 3T3 host cells following 48 hours exposure, and were identified as putative *T*. *cruzi* cytochrome P450 (TcCYP51) inhibitors. TcCYP51 inhibitors are not favourable for the drug discovery pipeline for treatment of Chagas Disease infection due to clinical and pre-clinical failures. To determine if there were *in vitro* inhibitory characteristics of these compounds that could aid the prediction of TcCYP51 inhibition further profiling using imaging and fluorescence based assays was undertaken. It was determined that *in vitro* profiles, coupled with analysis of chemical structure, could support the early prediction of putative TcCYP51 activity and thus enable early de-prioritisation of these compounds from progression through the drug discovery pipeline.

## Introduction

Chagas disease, caused by *Trypanosoma cruzi* is responsible for around 14 000 deaths per year, with a further 6–8 million people effected by the disease^1^. There are two current drugs, benznidazole (BZ) and nifurtimox (NFX), used to treat infection with this parasite, which require complicated and lengthy dosing regimens and present associated side effects which compromise compliance^2,3^. In addition, these drugs have questionable efficacy, particularly associated with the chronic phase of the disease^4^. The need for new drugs drives early discovery programmes to identify compounds with new modes of action against the parasite.

We have recently developed an image-based technique, incorporating the fluorescent labels, Hoechst and HCS CellMask Green™ to enumerate 3T3 fibroblasts infected with *T*. *cruzi* amastigotes following compound exposure^5^. From a high-throughput screening campaign targeting *T*. *cruzi*, new inhibitors from a diverse library of 25 000 compounds were identified with <10 µM activity against the parasite and a selectivity index (SI) of ≥10 fold to mammalian cells. A common feature observed with these compounds was the inability to clear parasites from host cells at the maximum response achievable (E_max_), following 48 hours incubation. We have previously observed that azole antifungals, including clotrimazole and posaconazole (POSA), also did not completely clear parasites from host cells^5^. A sub-efficacious effect had also been observed by other researchers utilising image-based technology to assess the activity of known TcCYP51 inhibitors against *T*. *cruzi*^6^. Consequently, the compounds identified were tested against the recombinant TcCYP51 enzyme^7^, and shown to be putative inhibitors. The phenotypic characteristics of *T*. *cruzi* infected cells following 48 hours treatment with these hit compounds strongly supported the prediction that a sub-efficacious effect was common to TcCYP51 inhibitors.

To determine if a more detailed inhibitory profile of these compounds could collectively aid rapid identification of TcCYP51 activity, we optimised image-based assays to enable temporal analysis of infected cells post-treatment. Compound activity following 24 and 96 hours incubation was assessed in addition to the impact of removal of compound pressure after 48 hours incubation. The *in vitro* profiles of the hit compounds were compared to POSA and the nitro-heterocyclic drugs used to treat Chagas disease, NFX and BZ. The mode of action of NFX is suggested to involve generation of oxygen radicals causing oxidative stress in *Trypanosoma brucei brucei* parasites^8^. Although there may be other modes of action involved with NFX inhibition of *T*. *cruzi*^9^, type 1 nitro-reductase activation in *T*.*b*. *brucei* supports oxidative stress as the likely mode of action of this compound. Clemastine fumarate (CF), identified by us and other researchers to be active against *T*. *cruz*i^5,10^, with an unknown mode of action (MOA), was also tested to determine if CF was active against recombinant TcCYP51.

It has been shown that *T*. *cruzi* life cycle stages have different amounts and compositions of sterol classes. Amastigotes have a simple sterol biosynthetic pathway, lacking some of the reductases found in epimastigotes. As a result, multiplying *T*. *cruzi* amastigotes do not form a number of endogenous sterols, the major neutral lipids of epimastigotes^11^. Due to a smaller pool of endogenous sterols, it is reported that TcCYP51 inhibitors display ~ 100 fold difference in activity between these life cycle forms^11^, however the activity of larger collections of TcCYP51 inhibitors against amastigote and epimastigotes has not been reported. We developed an assay to determine compound activity against epimastigotes, using PrestoBlue^®^, a reagent novel for this purpose. A direct comparison of compounds, newly identified as TcCYP51 inhibitors, between *T*. *cruzi* Tulahuen strain intracellular amastigotes and epimastigotes was undertaken.

The chemical structures of *T*. *cruzi* TcCYP51 inhibitors are quite varied and there is not necessarily one type of functional group responsible for activity against the enzyme^12^. However, some chemical classes are commonly associated with specific inhibition, including pyridine, pyrimidine and azole derivatives^13^. The pyridyl groups of the pyridine-based inhibitors UDO and UDD have been shown to coordinate with the heme group of the TcCYP51 enzyme^14^. Two- and 4-pyridyl motifs have also been associated with anti-Tc CYP51 activity^7,14–16^. The structures of the identified hit compounds in these studies were compared to motifs with TcCYP51 activity. Collectively, the *in vitro* inhibitory profiles of the hit compounds, in combination with their chemical structures were investigated as tools to predict putative TcCYP51 activity.

## Methods

### Maintenance of *T*. *cruzi* parasites

*T*. *cruzi* Tulahuen strain epimastigotes were kindly provided by Professor Frederick Buckner (University of Washington, USA), and were differentiated into metacyclic trypomastigotes in liquid media in artificial bug urine, TAU3AAG^17^. The mammalian stages of *T*. *cruzi* were maintained in 3T3 mouse embryonic fibroblasts (ATCC, CCL92) at a multiplicity of infection (MOI) of 10:1, as previously described^5^. Egressed *T*. *cruzi* trypomastigotes were harvested from the supernatant of infected host cells four days post infection, for the use in experiments or continuation of the parasite culture.

*T*. *cruzi* epimastigotes were cultured in liver infusion tryptose (LIT) media^18^, supplemented with 10% FBS and 1% penicillin streptomycin. Cultures were incubated at 28 °C and 5% CO_2_. Cultures were passaged in the logarithmic growth phase by sub-culturing every five days, at a density of 6.25 × 10^3^ parasites/mL; or three days, at a density of 1.20 × 10^6^ parasites/mL.

### Maintenance of mammalian cells

3T3 cells were sub-cultured every 2 or 3 days at concentrations of 2.00 × 10^5^ or 4.00 × 10^5^ cells per 175 cm^2^ flask, respectively. Cells were not used past passage 7 due to loss of contact inhibition at higher passages. Cells were grown in RPMI in with no phenol red (Life Technologies, USA), supplemented with 10% FCS at 37 °C and 5% CO_2_ in a humidified environment. HEK293 cells were cultured in RPMI every 3 or 4 days at concentrations of 1.00 × 10^6^ or 1.20 × 10^6^ cells per 175 cm flask, respectively. Cells were grown in RPMI supplemented with 10% FCS at 37 °C and 5% CO_2_ under humidified conditions.

### Compounds

NFX was isolated from Lampit tablets by Dr Agatha Garavales. BZ was provided by Epichem Pty Ltd, POSA was purchased from Sigma (USA) and CF from Tocris Bioscience (USA). Solid samples of the active compounds **1–5** were purchased from Chemdiv (Chemdiv, USA). **Compound 1** = 1-(2-Chlorobenzyl)-3-(4-ethylphenyl)-1-(3-pyridinylmethyl)urea; **compound 2** = Ethyl oxo[2-(4-phenyl-1-piperazinyl)-2-(3-pyridinyl)ethyl]amino}acetate; **compound 3** = 1-(1-Acetyl-4-piperidinyl)-3-(4-chlorophenyl)-1-(3-pyridinylmethyl)urea; **compound 4 = **3-(4-Ethoxyphenyl)-1-(4-methylbenzyl)-1-(3-pyridinylmethyl)urea; **compound 5** = 3-(3,4-Dichlorophenyl)-1-(4-methylbenzyl)-1-(3-pyridinylmethyl)urea.

### Compound handling

Compounds were added to assay plates using a Minitrak™ compound handling system (PerkinElmer, USA) by preparing an intermediate dilution of compounds. One microlitre of compound in 100% DMSO was added to 20 µL of filtered H_2_O (Milli-Q® Purification System, Merck Millipore, USA). Five µL of this dilution was added to the wells of 384-well plates.

Control compounds used to assess the sensitivity of the *T*. *cruzi* amastigote assay were NFX, BZ and POSA. To calculate compound activity, 12.0 µM NFX was used as a positive control and 0.366% DMSO was used as a negative control. To determine the activity against the host cell, 30.0 µM of puromycin was used as a positive control. The percentage normalised activity was determined using the equation outlined in our previous publication^19^ and the reproducibility of the assay was determined using the Z’-factor^20^.

### *T*. *cruzi* image-based assay

The assay was performed as previously described^5^. Briefly, 3T3 cells were seeded at a concentration of 1 × 10^3^ cells in 50 µL per well into collagen I coated 384-well plates (PerkinElmer, USA). Following 24 hours incubation at 37 °C and 5% CO_2_, 10 µL of parasites were added to wells at a multiplicity of infection (MOI) of 5:1 (parasites: host cells) with a Multidrop™ dispenser (Thermo Fisher, USA). Following 24 hours incubation, wells were washed with PBS (containing Mg^2+^ and Ca^2+^) to remove uninfected parasites. One wash was undertaken by using a Multidrop plate dispenser and two washes with a Bravo liquid handling device (Agilent Technologies, USA). Five microlitres of compound were added to plates and incubated for 48 hours. Cells were then fixed and stained with Hoechst (Life Technologies, USA) and HCS CellMask™ Green (Life Technologies, USA) and image acquisition was performed an Opera™ High Content Screening System (PerkinElmer, USA) at 20 times magnification. The positive control was 12.0 µM NFX and the negative control was 0.366% DMSO.

### HEK293 assay

The HEK293 assay was undertaken as described previously in a 48 hour assay^5^. Briefly, HEK293 cells were seeded into 384-well clear/black sterile tissue culture treated plates (Greiner, Austria) at a concentration of 7.27 × 10^4^ cells/mL. Plates were incubated for 24 hours before the addition of compounds. After 48 hours incubation in the presence of compounds, 10 µL of resazurin was added to wells, and incubated for 3 hours. Plates were read on an Envision plate reader (PerkinElmer, USA) at ex/em 530/595. The positive control was 32.5 µM puromycin and the negative control was 0.433% DMSO, slightly different to the *T*. *cruzi* assay due to the change in volume, whilst utilising the same stock control plate. For 120 hours incubation, 4.54 × 10^3^ cells/mL were used as an initial inoculum.

### Primary screening

The primary screening collection of 24 993 compounds optimised for lead like properties was contained in 71 × 384-well polypropylene plates (Corning, NY, USA). The compounds were housed at ***Compounds Australia***, Brisbane, Australia. The final concentration of compounds in the *T*. *cruzi* amastigote assay was 4.00 µM.

For the identification of active compounds, the mean % activity plus 3 standard deviations of the collection was applied. The maximum activity achievable (E_max_) of compounds was determined by taking (or as close to, if concentrations in the E_max_ were limited) the percentage activity at the mid concentration within the plateau of activity in the concentration response curve (see Supplemental Fig. 1, example NFX).

### Retest

Compounds considered selectively active against *T*. *cruzi* displayed an IC_50_ value ≤10.0 µM and a selectivity of ≥10.0 to 3T3 host cells and human embryonic kidney (HEK293) cells. This activity cut off is the basis for a target product profile (TPP) for *T*. *cruzi* by the Drugs for Neglected Diseases Initiative (DND*i*)^21^.

To determine the IC_50_ values of active compounds, DMSO samples were manually cherry picked from primary screening plates and diluted out in a log series of concentrations. Final assay concentrations of compounds ranged from 4.03 µM to 4.03 × 10^−3^ µM. The cytotoxicity of compounds was determined against HEK293 cells. As this assay had a slightly different end point volume, final compound concentrations ranged from 4.76 µM to 4.76 × 10^−3^ µM. A selection of 17 analogues and confirmed active compounds were tested at a final concentration range of 18.3 µM to 1.83 × 10^−4^ µM from compounds in DMSO stored in tube samples against *T*. *cruzi*.

Compounds active against *T*. *cruzi* were purchased as solid samples for confirmation of activity and tested over n of 4 replicates. Stocks were serially diluted in log concentrations (in DMSO) to give final assay concentrations ranging from 73.3 µM to 1.83 × 10^−3^ µM. Compound **1** was further solubilised to a final concentration 36.6 µM in the assay as some insolubility was observed in the intermediate dilution in water. This was only undertaken for the amastigote assay. Dilutions were prepared with a multichannel pipette (Finpipette™, Thermo Fisher Scientific, USA) in 384-well polypropylene plates (Corning, NY, USA). Compounds were tested against HEK293 cells (n of 2 replicates) to assess the potential cytotoxicity against replicating mammalian cells following 120 hours exposure.

### Further profiling of *T*. *cruzi* selectively active compounds

#### 24 hour image-based assay

Compounds were added to assay plates as described for the 48 hour assay, although incubation time was decreased to 24 hours. Seven fields were imaged as in the 48 hour assay, although due to a lower level of infection, the Z’-factor of the assay was not acceptable (<0.5)^20^. The number of fields acquired was increased to 14 fields per well to obtain a Z’-factor of >0.5. Two experimental replicates were performed.

#### 96 hour image-based assay

The assay format was the same as the 48 hour assay, although plates were incubated for 96 hours. Following this length of incubation it was observed that parasites had egressed from host cells, as we have previously observed^5^. Two extra wash steps were included to remove as many extracellular parasites as possible to reduce impact on determination of intracellular parasites. Due to the level of egress in negative control wells, the number of infected cells was estimated as previously^5^. Two experimental replicates were undertaken.

### Wash-off assay to assess parasite survival following compound removal

The *T*. *cruzi* assay 48 hour assay was performed. Following, plates were washed 5 times with PBS (containing Mg^2+^ and Ca^2+^) on a Bravo liquid handler to remove compound pressure. Plates were incubated for an additional 72 hours and wells were washed 5 times before fluorescent labels were added. Plates were imaged on an Opera confocal-based high-content system with 7 fields per well acquired, at 20 times magnification. As considerable parasite egress was observed, only E_max_ concentrations were used for analysis. Four experimental replicates were undertaken.

### Isolation of intracellular amastigotes and determination of viability by differentiation

Final concentrations of 1.00 µM POSA and 12.0 µM of NFX were added to *T*. *cruzi* infected 3T3 cells following the same protocol for the 48 hour assay. Compounds were added to one column each, over four plates. Following either 48 hours or 96 hours exposure of *T*. *cruzi* infected 3T3 cells, media was removed in two plates and wells washed three times with PBS using a multichannel pipette. Following compound treatment, no egress of trypomastigotes was observed. Fifty microlitres of Accutase® (ThermoFisher, USA) was added wells and incubated for 15 minutes at 37 °C and 5% CO_2_. Host cells were collected by centrifuging at 3000 g for 8 minutes. The cell mixture was passed through a 26 ½ gauge needle to incite host cell damage^22^, and agitated using a vortex mixer before being centrifuged again at 300 g for 5 minutes. This process was repeated before suspension in 5 mL of LIT in a 25 cm^2^ flask. The cell mixture was incubated at 37 °C 5% CO_2_ for 24 hours before centrifugation at 300 g for 8 minutes in an effort to remove some of the host debris remaining. The pellet was suspended in 10 mL of fresh LIT media, and incubated in a 25 cm^2^ flask at 28 °C, in a normal environment. After 5 weeks in culture the media was refreshed and cells incubated for a further 5 weeks. By this stage, isolated amastigotes had differentiated into epimastigotes, which were enumerated.

### 120 hour *T*. *cruzi* epimastigote assay

Epimastigotes in the logarithmic phase of growth were dispensed into black/clear tissue culture treated 384-well plates (Greiner, Austria) at a density of 3.10 × 10^3^ parasites/mL, in 50 µL of LIT. Additions were made with a Multidrop dispenser and compounds were added directly following parasite addition. Plates were incubated for 112 hours before the addition 10 µL of PrestoBlue^®^ viability reagent (Thermo Fisher, USA). Plates were incubated for 8 hours before determination of the fluorescent signal on an Envision multilabel reader (PerkinElmer, USA) ex/em 530/595. The positive control was NFX at a final concentration of 14.20 µM and 0.433% DMSO as a negative control.

### Compound activity against the recombinant Tc CYP51 enzyme

The activity of compounds against TcCYP51 was determined at the University of Dundee as outlined previously^7^. This assay utilises the fluorogenic probe benzyloxymethyloxycyanocoumarin (BOMCC), activated by TcCYP51. Initially the assay was performed with one experimental replicate for each test compound and 5 replicates for POSA, as a positive control. Three replicates per compound were used to confirm activity, with 5 replicates of POSA. Preliminarily testing of MMV001239 was performed using this assay, with 1 replicate concentration response curve generated to establish whether it was a putative TcCYP51 inhibitor.

### Statistical analysis

Compound IC_50_ values in the *T*. *cruzi* and HEK293 cell-based assays were determined using Prism 5.0 (GraphPad software), derived by a sigmoidal concentration response (variable slope) analysis. Compound IC_50_ values in the TcCYP51 *in vitro* assay were calculated in GraFit (Erithacus software) by non-linear slope analysis. All data are presented as mean ± standard deviation. The significance of % clearance of parasites at compound E_max_ concentrations was calculated in Prism 5.0, using an unpaired student’s t- test. Compound activities with p-values of <0.05 were considered to be significantly different from controls.

### Data availability

The datasets generated during and/or analysed during the current study are available from the corresponding author on reasonable request.

## Results

### Primary screening

**Figure 1:**
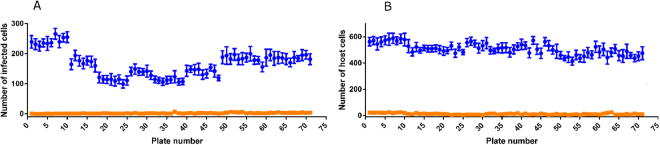
Controls in the *T*. *cruzi* infected 3T3 host cell image-based assay to identify inhibitors against the *T*. *cruzi* parasite during single concentration primary screening of a collection of 24 993 synthetic compounds. The values were averaged from 14 wells each for positive and negative controls in 384-well plates. The values are shown for the controls within each plate in the primary screening campaign. (**A**) The number of *T*. *cruzi* infected cells (**B**) the number of 3T3 host cells, incubated in the presence of 0.366% DMSO (negative control) and 12.0 µM nifurtimox (positive control).

**Figure 2:**
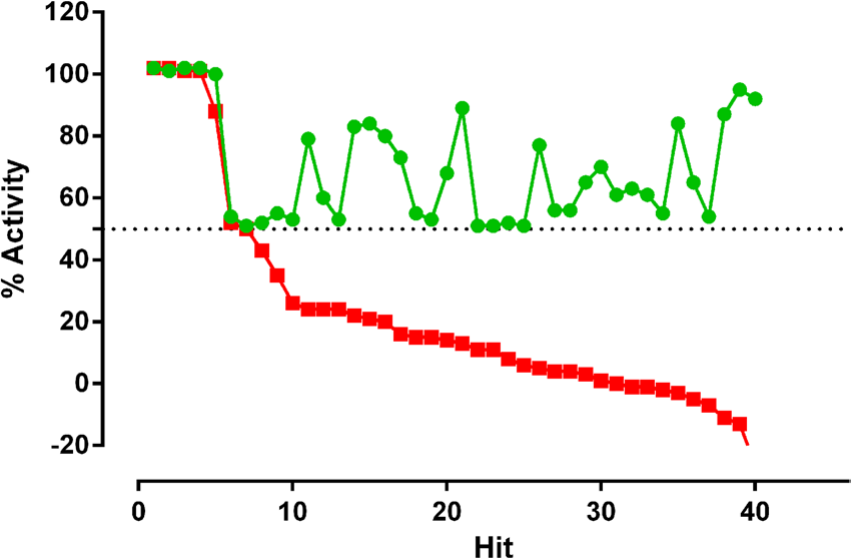
The activity of 40 hit compounds identified from screening a collection of synthetic compounds optimised for lead-like properties against *T*. *cruzi* amastigotes using an image-based assay, at a concentration of 1.10 µM. The activity of compounds is shown against *T*. *cruzi* and 3T3 host cells from the same assay. Green = % of *T*. *cruzi* infected host cells in relation to nifurtimox, red = % 3T3 host cells in relation to puromycin, following 48 hours exposure to compounds,  respectively.

The cut off of activity applied to select active compounds was 50% against *T*. *cruzi* parasites. The mean parasite number was 166 ± 42.9 for the positive control and 2.19 ± 1.52 for the negative control, with a mean signal window of 139. The numbers of parasites and host cells identified are shown in Fig. 1. Forty hits were discovered with ≥50% activity against *T*. *cruzi*, with varying levels of activity against 3T3 host cells (Fig. 2). The robustness of the assay was illustrated by a mean Z’-factor of 0.678 for the amastigote assay and 0.764 for 3T3 host cells.

### Retest

Twenty-four of the 40 hit compounds cherry-picked from the primary screening collection reconfirmed with activity ≥50% at the top screening concentration of 4.00 µM, a confirmation rate of 60.0%. Five compounds had IC_50_ values ≤10 µM and selectivity indices (SI) from a ratio of the 3T3 host cell/parasite activity ≥10. IC_50_ values ranged from 0.293–1.96 µM against the parasite. These compounds did not display cytotoxicty against HEK293 cells up to a final concentration of 4.37 µM.

Fourteen compounds, consisting of 5 original hits and selected analogues, were tested from DMSO stocks stored in tube samples to determine IC_50_ values against *T*. *cruzi*. Three of the original hit compounds exhibited activity at expected levels (**compounds 1–3**), whilst two of the original hits did not re-confirm activity at any of the concentrations screened. Two analogues, compound **4** and compound **5**, displayed IC_50_ values of 1.40 ± 0.0799 µM and 1.80 ± 0.00354 µM, respectively. All of these compounds exhibited sub-efficacious activity at the plateau of the curve (the E_max_), with 70.5–84.5% inhibition, similar to the effect exhibited by POSA, with 70.0% inhibition at the E_max_. Compounds **1–5** did not remove the entire parasite population, unlike NFX and BZ, which killed 100% of the parasite population at E_max_ concentrations. The IC_50_ values for compounds **1–5** and the percentage of parasites present following 48 hours incubation are shown in Table 1. These compounds all contain a pendent 3-pyridyl motif and **1**, **5** and **4** are related analogues.

**Table 1:** IC_50_ values, selectivity indices (SI) and % activity displayed at the E_max_ for the 5 hit compounds identified from the screening campaign, and retested from DMSO stocks. The activity of the control compounds benznidazole (BZ), nifurtimox (NFX), posaconazole (POSA) and puromycin (PURO) are included. Clemastine fumarate (CF) was screened alongside the temporal experiments, as activity was previously determined in the image-based assay^5^ and this activity is shown for comparison. SI for all compounds is in relation to 3T3 cells. As there was no activity displayed against 3T3 cells for compounds **1–5**, the SI was calculated from the IC_50_ value being >18.3 µM. In addition, for the controls >127 µM for NFX and BZ; 1.00 µM for POSA and >26.0 µM for CF. For compound **1**, the E_max_ is only just reached and is from the first point at the plateau of activity. All compounds were screened from samples stored in DMSO, except for CF which was prepared from solid sample and data was taken from the temporal experiments. NS = not selective to the parasite therefore the E_max_ is not quoted. Figures are rounded to 3 significant figures.

Name	Structure	IC_50_ *T*. *cruzi* (µM)	SI to 3T3^1^	% activity (E_max_)
1		3.48 ± 0.622	>5.26	84.5 ± 6.36
2		2.17 ± 0.499	>8.43	76.5 ± 3.54
3		1.29 ± 0.368	>14.2	70.5 ± 0.707
4		1.40 ± 0.0799	>13.1	76.0 ± 1.41
5		1.82 ± 0.00354	>10.0	88.0 ± 9.90
BZ		3.56 ± 2.339	>35.7	99.5 ± 2.12
NFX		0.709 ± 0.161	>179	102 ± 1.41
POSA		0.00272 ± 0.00873	>368	70.0 ± 8.49
PURO		2.01 ± 0.329	1.36	NS
CF		0.570 ± 0.0396	>45.6	96.8±1.26

### Solid stock confirmation of activity

**Figure 3:**
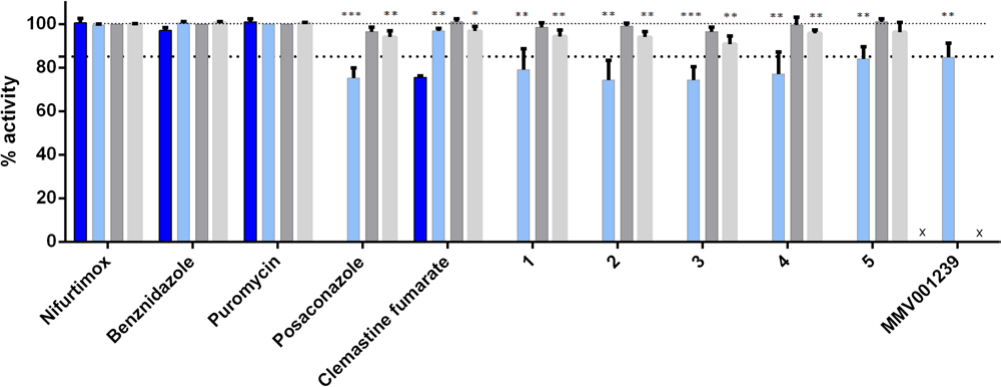
Percentage activity at E_max_ concentrations of hit compounds **1–5**, clemastine fumarate and reference compounds against *T*. *cruzi* intracellular parasites, following incubation for either 48, 96 hours, or following a wash-off assay. *p < 0.05; **p < 0.005, ***p < 0.0005. Percentage activity at the E_max_ for compound MMV001239 is shown following 48 hours incubation. Error bars represent the standard deviation of measurements. X = experiment not performed. Dark blue bars=24 hour assay, light blue=48 hour assay, dark grey=96 hour assay, light grey=wash-off assay.

Compounds were purchased as solid stocks and the IC_50_ values determined against *T*. *cruzi* amastigotes. All compounds reconfirmed activity with less than a 3 fold difference in IC_50_ values compared to the DMSO stock for n of 4 independent replicates. These compounds displayed E_max_ values ranging from 73.5–92.2%, whilst BZ and NFX reproducibly resulted in clearance of parasite from 100% of infected host cells at E_max_ concentrations. MMV001239, predicted to be a CYP51 inhibitor from our previous testing^5^ was tested again in the *T*. *cruzi* 48 hour assay to obtain the E_max_ for a further two replicates, thus four replicates in total. MMV001239 displayed sub-efficacious activity of 85 ± 6.4% (Fig. 3), with significance displayed (p = 0.00390).

### Temporal activity of compounds

Compounds **1–5**, CF and control compounds puromycin, POSA, BZ and NFX were tested against *T*. *cruzi* parasites following 24, 48, and 96 hours exposure and additionally in a compound wash-off assay. Small populations of parasites remained following 48 hours incubation and after wash-off of compounds **1–5**. Therefore, two additional experiments were undertaken (n of 4 replicates total) to determine if the data for these assays could be used to provisionally identify TcCYP51 inhibitors. For 24 and 96 hour incubations, n of 2 experimental replicates were performed.

It was not possible to estimate an IC_50_ value for compounds **1–5** following 24 hours incubation, however, 84.5% and 83.5% activity was observed for compounds **1** and **5**, respectively, at the highest concentration of 36.6 µM and 73.3 µM, respectively. The IC_50_ value obtained for CF was 1.09 ± 0.0540 µM, with 75.5% activity at the E_max_. NFX and BZ could have an IC_50_ value attributed to them (1.20 ± 0.0213 and 7.56 ± 2.37 µM, respectively), with 100% activity at E_max_ concentrations. POSA however showed a plateau with a mean activity of < 50% and thus an IC_50_ was not determined.

The percentage activity at the E_max_ for compounds **1–5** in each assay (24, 48, 96 hours and wash-off) are shown in Fig. 3. The mean activity at E_max_ concentrations following 48 hours incubation ranged from 74.2–84.0% for the 5 hit compounds and 96.8% for CF. Treatment with BZ or NFX resulted in 100% of infected cells cleared of parasite. Exposure to POSA for 48 hours resulted in 75.3% activity at the E_max_. The statistical significance (p-value cut off) of inhibition at the E_max_ are highlighted in Fig. 3. In relation to the E_max_ activity of NFX, all five hits demonstrated a significantly reduced efficacy, with p-values of <0.000200 to 0.00500. POSA was found to be significantly different from NFX with a p-value of <0.000100. In contrast, CF displayed increased efficacy after 48 hours incubation (96.8%), with a p-value of 0.00730.

**Figure 4:**
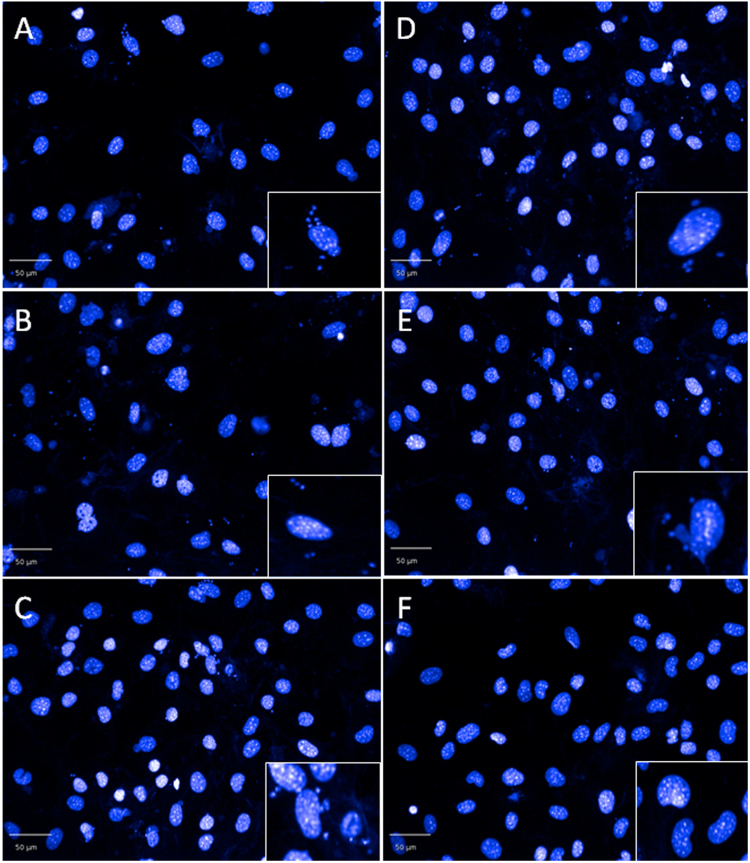
Residual *T*. *cruzi* intracellular amastigotes following 48 hours incubation with compounds **1–5** and nifurtimox, followed by wash-off of compound and a further 72 hours incubation in the absence of compound. (**A**–**E**) = compounds **1–5** respectively at E_max_ concentrations. (**F**) 12 µM nifurtimox.

After 48 hours incubation, followed by wash-off, and an additional incubation of 72 hours, the mean percentage activity was 90.0–96.3% for compounds **1**–**5**. NFX and BZ cleared parasites from 100% of infected cells, whereas POSA only caused 94.0% reduction in the number of infected cells. CF reduced parasite numbers by 96.8% during the same time period. A significant difference in the efficacy, compared to NFX, was observed for compounds **1–4**, with p -values ranging from 0.00230–0.00530. In all cases, parasites could be seen in the host cells (Fig. 4). Parasite re-infection of new host cells following wash-off was not evident when compared to data obtained after 48 hours incubation.

Exposure to hit compounds for 96 hours resulted in 98.5–101% reduction in infected cells and none were significantly different from NFX. Exposure to POSA for 96 hours resulted in 96.5% efficacy. Whilst there was no difference in activity for NFX and BZ, there was an increase in activity from 48–96 hours incubation for compounds **1–5**, ranging from 17.0–24.8%. POSA treatment resulted in increased activity at the E_max_ by 21.3%, whilst CF showed an increase in the E_max_ of 4.30%.

### Activity of compounds against epimastigotes

An IC_50_ value could not be estimated for hit compounds **1–5** following 120 hours exposure to *T*. *cruzi* epimastigotes, although could be for CF. Compounds **3 ** and **5** displayed > 50% activity at the highest dose of 86.6 µM. Table 2 shows the activity of hit and control compounds following 120 hours incubation with either *T*. *cruzi* epimastigotes or HEK293. Whilst compounds **1–4** showed < 50% activity against HEK293 cells, 94.0% and 99.5% activity was exhibited by compound **5** and CF, at the highest screening doses of 79.4 µM and 28.2 µM, respectively.

**Table 2:** IC_50_ values for the 5 hit compounds identified from the screening campaign, along with clemastine fumarate (CF) against *T*. *cruzi* epimastigotes following 120 hours exposure. The control compounds were benznidazole (BZ), nifurtimox (NFX), posaconazole (POSA) and puromycin (PURO). SI is in relation to HEK293 cells. 1. Cannot define a robust SI as 99.5% activity against HEK293 cells, similar to the activity at the highest concentration against epimastigotes (97.0%). NA= SI cannot be determined as an IC_50_ value could not be attained as no plateau of activity. % activity is stated if the compound was >50% active against the parasites/HEK293 at the maximum screening concentration. Figures are rounded to three significant figures.

Compound	IC_50_ value (µM)	SI to HEK293 cells
1	83.0% at 86.6 µM	NA at 79.4 µM
2	NA at 86.6 µM	NA at 79.4 µM
3	NA at 86.6 µM	NA at 79.4 µM
4	NA at 86.6 µM	NA at 79.4 µM
5	91.0% at 86.6 µM	94.0% at 79.4 µM
Nifurtimox	1.11 ± 0.131	>136
Benznidazole	5.78 ± 0.477	>26.1
Puromycin	0.566 ± 0.141	0.386
Clemastine fumarate	6.40 ± 0.0217	99.5% at 28.2 µM^1^

### Compound activity against the recombinant Tc CYP51 enzyme

**Figure 5:**
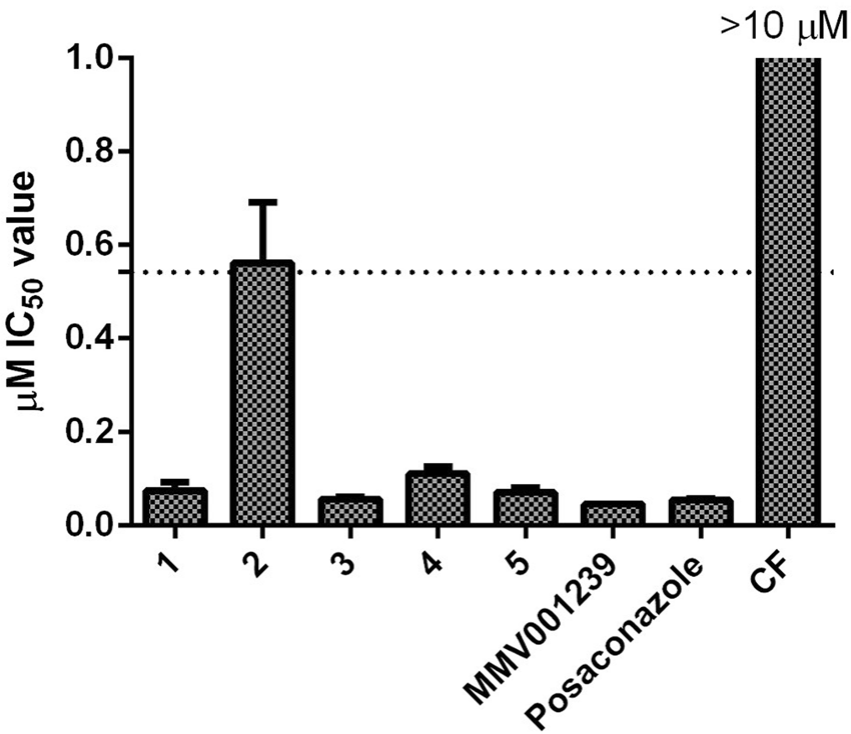
Activity of the five hit compounds, clemastine fumarate (CF) and posaconazole against *T*. *cruzi* recombinant CYP51. The activity is taken from N = 2 experiments. The IC_50_ value of POSA was 0.0543 ± 0.00387 µM as a control TcCYP51 inhibitor.

Figure 5 shows the activity of compounds **1**–**5**, CF and the reference TcCYP51 inhibitor POSA against TcCYP51. The cut off value to define an inhibitor of TcCYP51 was at least a log difference in activity over POSA to give confidence that the predominant mode of action is not CYP51 driven^7^. All five hit compounds were shown to be putative TcCYP51 inhibitors, with ≥a 10 fold difference between the IC_50_ value determined for POSA. Compound **2** showed a mean of 0.562 ± 0.130 µM against TcCYP51, which was not significantly different from 10 fold the POSA activity, with an IC_50_ value of 0.543 ± 0.00387 µM. This was similar to the inhibition demonstrated by the known TcCYP51 inhibitor fluconazole, reported in this assay previously (0.88 µM)^7^.

MMV001239 was defined as a potential TcCYP51inhibitor following *in vitro* testing, with clearance of 84.8% of parasites. From a single concentration response curve, this compound displayed activity with an IC_50_ value of 0.0460 µM against recombinant TcCYP51. CF was not determined to be a TcCYP51 inhibitor, with an IC_50_ value of ≥10 µM against the recombinant enzyme.

### Isolation, differentiation and growth of epimastigotes from host cells treated with POSA for 48 and 96 hours

**Figure 6:**
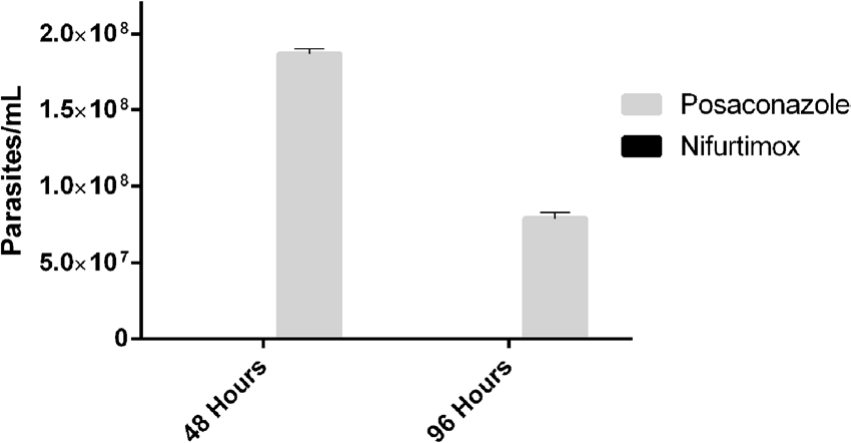
Survival of isolated intracellular *T*. *cruzi* amastigotes following exposure to either posaconazole or nifurtimox for 48 hours, followed by differentiation and growth in liquid medium. Amastigotes were isolated from infected 3T3 cells following treatment with either 1.00 µM of posaconazole or 12.0 µM of nifurtimox for 48 hours. Parasites were differentiated in LIT and grown for 5 weeks until in the log phase of growth before enumerating.

To determine if the *T*. *cruzi* parasites within infected cells, remaining after POSA treatment for 48 and 96 hours, were viable, amastigotes were mechanically isolated from cells, differentiated to epimastigotes and grown in liquid medium. Figure 6 shows the number of epimastigotes present following an extended incubation in which cells had reached a log phase of growth. There were no epimastigotes detected following treatment of amastigotes with 12.0 µM of NFX.

### *In vitro* characteristics to identify TcCYP51 inhibitors

Table 3 shows characteristics that can be used to identify putative Tc CYP51 inhibitors determined by *in vitro* characteristics exhibited by the TcCYP51 inhibitors compounds **1**–**5**, MMV001239 and POSA compared to NFX, BZ and CF in *T*. *cruzi* image and fluorescence-based assays.

**Table 3:** *In vitro* characteristics that can be used to define TcCYP51 inhibitors based upon data from profiling the Tulahuen strain against the 5 hit TcCYP51 inhibitors, plus the control TcCYP51 inhibitor, posaconazole. ≤90% was used as a cut off as this was the highest inhibition for the 5 test compounds during temporal testing. Following 48 hours incubation, 5/5 CYP3/TcCYP51 inhibitors showed a significant difference (p < 0.05) in efficacy compared to nifurtimox, following 48 hours incubation and 4/5 in the wash-off assay, therefore an increase in significance would improve confidence. NA = not active (IC_50_ value >10 µM).

*In vitro* incubation/life cycle	Efficacy	Activity (µM IC_50_)
24 hours amastigote	NA	>10
48 hours amastigote	≤90% (p < 0.05)	<10
96 hours amastigote	≥15% difference (to 48 hours). No significant difference to NFX	<10
48 hours/wash/72 hours amastigote	>96% (p < 0.05) No out-growth of parasites	<10
120 hours epimastigote	NA	>10

## Discussion

CYP51 was considered to be an important target in *T*. *cruzi* for some time because of the potency demonstrated *in vitro* by a number of CYP51 inhibitors, and suppression exhibited *in vivo*^12,13,16,23^. However, more recent investigations have highlighted the issues with CYP inhibitors used to target *T*. *cruzi* in pre-clinical and clinical studies. Failure of POSA and ravuconazole (E1124) to treat chronic Chagas infection^24,25^, combined with poor absorption of POSA demonstrated during clinical trials for other disease indications, have raised concerns, as well as provided insights to explain these failures^26^.

Forty-two % of the hits identified from screening a large GSK library against *T*. *cruzi* were found to be putative TcCYP51 inhibitors^27^. We have also found that 64% of the active compounds we identified from screening a library containing FDA approved compounds and lead candidates, were TcCYP inhibitors^5,28^. Hence, these represent a significant portion of the hit molecules identified through screening. The consensus of most laboratories to deprioritise CYP inhibitors is further highlighted by the Drugs for Neglected Diseases Initiative’s (DND*i*) decision not to pursue this class of inhibitor^29^. As a result, the need to be able to readily identify and deprioritise TcCYP51 inhibitors is imperative in *T*. *cruzi* drug discovery to avoid pursuing compounds that are high risk for development.

Using high content imaging, we have previously demonstrated that known CYP51 inhibitors, clotrimazole and POSA did not completely remove *T*. *cruzi* amastigotes from host cells following 48 hours incubation^5^. We recently identified five new compounds (**1–5**) with novel *T*. *cruzi* activity, demonstrated to be putative TcCYP51 inhibitors. A comparison of the activity at the E_max_ of these compounds was made to other molecules with a mode of action not associated with CYP51. This collection consisted of drugs used to treat Chagas disease, NFX, BZ and additionally the known *T*. *cruzi* inhibitor CF, which did not inhibit recombinant TcCYP51 in these studies. Compounds were also compared the known TcCYP51 inhibitor POSA. Compounds **1–5** failed to clear all parasites from host cells following 48 hours incubation, a characteristic also shared by POSA and to a much lesser extent, CF. The ability of CF to clear the majority of parasites from host cells was similar to NFX and BZ, which resulted in no host cell infection. The reduced efficacy displayed by the TcCYP51 inhibitors was improved with increased exposure to 96 hours. With a lower exposure of 24 hours, an IC_50_ value could be determined for NFX, CF and BZ, however not the CYP51 inhibitors. Therefore, a slow-acting *in vitro* profile is indicative of TcCYP51 inhibition, providing an indicator to be used to identify this MOA.

Small populations of parasites still remained following 96 hours with POSA and growth of isolated parasites from host cells revealed that longer periods of time may be required to clear parasites. Similarly, it has recently been shown that following 96 hours exposure of *T*. *cruzi* infected peritoneal macrophages to POSA, parasites remaining are capable of replication and able to reinfect peritoneal macrophages^6^. Outgrowth studies over time with a larger, more chemically diverse collection of TcCYP51 inhibitors would be warranted to support the slow-acting nature of compounds with this MOA. Previous studies have shown that *T*. *cruzi* treated with POSA overexpress CYP51^30^ and this could contribute to parasites becoming refractive to treatment. Inhibition of replication (quiescence^31^) in the chronic phase of the disease could further suppress the growth of parasites, which may then be even more refractory to treatment. This may effectively contribute to the failure of TcCYP inhibitors in the clinic.

Previous HTS campaigns against *T*. *cruzi* parasites have not commonly used an initial 48 hour assay to assess the activity of compounds from large compound collections. Longer assay incubation periods are generally used (up to 7 days), which can bias results towards slow long- acting compounds, such as TcCYP51 inhibitors^32^. Assay design and technology must also be considered due to differences observed between some β-galactosidase and image-based assays^33^. Moraes *et al*. (2014) have demonstrated, utilising image-based technology, that a small collection of four known TcCYP51 inhibitors exhibited lack of efficacy following 48 hours incubation. Increased exposure of amastigotes to these TcCYP51 inhibitors for 96 hours resulted in an increase in parasite clearance^6^ which supports the criteria defined from the studies described herein.

A compound wash-off assay was used to ascertain whether parasite growth in the absence of compound pressure could be used to further support the *in vitro* identification of putative TcCYP51 inhibition. Four of the 5 newly identified TcCYP inhibitors, in addition to POSA resulted in a significant increase in the number of infected cells remaining in comparison to NFX. Small numbers of parasites remained at the E_max_ for all of the TcCYP51 inhibitors (3.60–9.80%), including POSA. Therefore, the continued presence of parasites following wash-off, with no outgrowth, could represent criteria for the identification of TcCYP51 inhibitors.

A novel assay to determine compound activity against the epimastigote life cycle stage of *T*. *cruzi* was used to build upon the *in vitro* profile of putative and known TcCYP51 inhibitors. IC_50_ values could not be determined for compounds **1–5**, nor POSA, however could be for NFX, BZ and CF. These results illustrate that a collection of putative and known TcCYP51 inhibitors demonstrate distinct differences in activity against *T*. *cruzi* epimastigotes in comparison to amastigotes. In addition, TcCYP inhibitors displayed less activity than the compounds tested with MOA’s unrelated to TcCYP inhibition. These criteria add to an *in vitro* profile to identify TcCYP51 inhibitors.

The chemical structure of a compound may suggest TcCYP51 inhibition as the mechanism of action. A screening campaign designed to identify mycobacterium inhibitors against TcCYP51 identified 4-pyridyl formamide motifs that bound with the *T*. *cruzi* crystal structure^16^. Two-^14^ and 4-^23^ pyridyl motifs are also associated with TcCYP51 activity of *T*. *cruzi* inhibitors. A CYP pharmacophore has been described as containing a basic heterocycle and two additional lipophilic or aromatic motifs in a trigonal or tetrahedral arrangement around a central atom^34^. The azole group is also a common motif that confers CYP51 activity^35^. The novel inhibitors identified in this study all contained a pendent 3-pyridyl motif, which is likely providing the readily accessible N-atom that interacts with the heme iron of the *T*. *cruzi* enzyme. In combination with the experimental data presented, this provides further suggests that these compounds are TcCYP51 inhibitors.

A combination of improved efficacy with increased exposure, the continued presence of parasites following wash-off with no out growth, a distinct activity profile against *T*. *cruzi* epimastigotes and a specific chemical structure contributes strongly to an *in vitro* profile supporting the identification of a TcCYP51 inhibitor. Taking into consideration the differences observed for the TcCYP51 inhibitors compared with non-CYP inhibitors studied in these studies, Table 3 shows the characteristics recommended for the identification of TcCYP51 inhibitors using a suite of *in vitro* high-throughput assays. This is the first time that statistical significance has been applied to efficacy to help define these criteria.

The profiles of compounds following 48 hours incubation with *T*. *cruzi*, in combination with structural characteristics, can aid prediction of TcCYP51 activity. We previously identified MMV001239 from the Medicines for Malaria Venture ***Malaria Box*** (MMV) as an inhibitor of *T*. *cruzi* intracellular parasites^5^. However, this compound did not clear all parasites from infected 3T3 fibroblasts. The presence of residual amastigotes, in addition to the presence of a pyridyl motif, suggested that this compound may be a TcCYP51 inhibitor, confirmed by *in vitro* testing against recombinant TcCYP51. Using spectrophotometric binding assays and X-ray crystallography MMV001239 was recently shown to be a TcCYP51 inhibitor revealing a binding site shared with other anti-trypanosomal compounds that target TcCyp51^36^. Utilising the criteria established here, such as efficacy and chemical structure, to predict TcCYP51 inhibitors could save time on more detailed studies, enabling immediate compound prioritisation.

We have applied the 48 hour assay criteria (≤90% inhibition) to identify four potential and two known TcCYP51 inhibitors from the Medicines for Malaria Venture (MMV) ***Pathogen Box***^37^. These include two fenarimol analogues, EPL-BS967 and EPL-BS1246, known inhibitors of TcCYP51^14^ and two known antifungals, biteranol, a triazol fungicide, and difenoconazol, shown *in silico* to bind to fungal CYP51^38^. These compounds are thus of limited interest to pursue, highlighting the success of this method. Remaining compounds contain azole and pyridyl groups, also supporting potential TcCYP51 inhibition. The application of high-content, high-throughput imaging in Chagas drug discovery is of great benefit to support the early identification of putative TcCYP51. De-prioritisation of inhibitors with activity against TcCYP51 support future identification of compounds with novel modes of action against the parasite.

## Electronic supplementary material

Supplementary Information
