# Supplementary material for: 3-pyridyl inhibitors with novel activity against Trypanosoma cruzi reveal in vitro profiles can aid prediction of putative cytochrome P450 inhibition
Source: Sci Rep. 2018 Mar 20;8:4901. doi: 10.1038/s41598-018-22043-z (PMC5861127; doi:10.1038/s41598-018-22043-z)

3-pyridyl inhibitors with novel activity against *Trypanosoma cruzi* reveal *in vitro* profiles can aid prediction of putative cytochrome P450 inhibition

Melissa L. Sykes[1*] and Vicky M. Avery[2]

1. Discovery Biology, Griffith Institute for Drug Discovery, Griffith University, Nathan, Queensland, Australia. Correspondence to m.sykes@griffith.edu.au
2. Discovery Biology, Griffith Institute for Drug Discovery, Griffith University, Nathan, Queensland, Australia.

Supplementary Figure 1. The criteria used to identify the E_{max} value of compounds. The E_{max} for each compound is at least two points in from the beginning of the plateau of activity (circled). The example compound in this graph is the reference drug, nifurtimox.

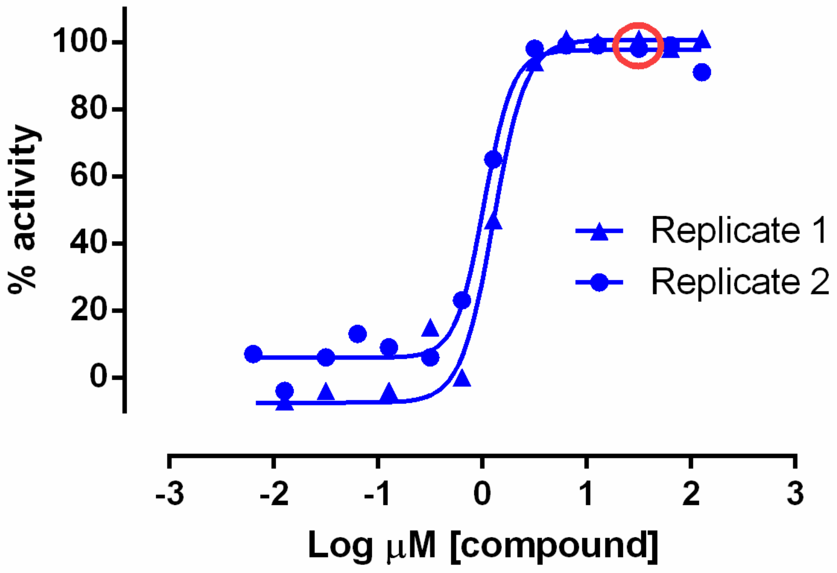

Supplement: Supplementary file 1 — Supplementary Information [file 41598_2018_22043_MOESM1_ESM.pdf]
